# Coherence Characteristics of Snow/Ice-Covered Areas Based on Space-Based Polarimetric Synthetic Aperture Radar Observations

**DOI:** 10.3390/s26113481

**Published:** 2026-06-01

**Authors:** Sang-Hoon Hong, Shimon Wdowinski, Seung-Kuk Lee

**Affiliations:** 1 Department of Geological Sciences, Pusan National University, Busan 46241, Republic of Korea; geoshong@pusan.ac.kr; 2 Institute of Environment, Department of Earth and Environment, Florida International University, Miami, FL 33199, USA; swdowins@fiu.edu; 3 Division of Earth and Environmental System Sciences, Pukyong National University, Busan 48513, Republic of Korea

**Keywords:** coherence, interferometric SAR, TerraSAR-X, polarization, X-band, snow/ice-covered areas

## Abstract

**Highlights:**

**What are the main findings?**

- The VV polarization in X-band synthetic aperture radar observations yields more coherent interferometric phase over snow- and ice-covered areas.
- L-band quad-pol InSAR observations provide similar coherence levels in both HH and VV polarizations.

**What are the implications of the main findings?**

- VV polarization is more suitable for X-band InSAR applications in cryospheric research.
- The design and application of space-based interferometric SAR missions for polar regions should take into account coherence characteristics associated with different radar frequencies.

**Abstract:**

Coherent space-based InSAR observations over snow- and ice-covered areas have been a valuable resource for cryospheric research. Coherence is considered a critical parameter for evaluating the quality of InSAR observations. This study evaluates the coherence characteristics of snow- and ice-covered areas using mainly fully polarimetric (quad-pol) X-band TerraSAR-X (TSX) and L-band ALOS PALSAR observations. The TSX data were acquired systematically during the Dual Receive Antenna campaign in 2010, while the quad-pol ALOS PALSAR L-band observations were acquired in 2007. A total of 57 TSX quad-pol images acquired over 17 areas at latitudes higher than 60° N were analyzed. The results across all study areas show relatively high coherence levels, ranging from 0.38 to 0.57, with the highest values observed in VV, followed by HH, and the lowest in HV. Interestingly, the highest coherence was found in the VV polarization, whereas HH coherence is typically higher than VV coherence in most InSAR applications. A comparative coherence analysis using quad-pol ALOS PALSAR L-band observations over selected snow- and ice-covered areas showed very similar coherence levels for both HH and VV polarizations. These results suggest that VV polarization is the most suitable for X-band InSAR applications over snow- and ice-covered areas.

## 1. Introduction

The Sixth Assessment Report (AR6) of the Intergovernmental Panel on Climate Change (IPCC) states that glaciers worldwide are continuing to retreat and shrink dramatically due to climate change. In the Arctic, climate change poses an increasing challenge not only to the natural environment but also to indigenous communities, threatening traditional ways of life and necessitating substantial investments for adaptation and, in some cases, relocation [1]. Furthermore, recent polar research has documented significant ice sheet mass loss and continuous glacier retreat in both Greenland and Antarctica [2,3,4]. In Greenland, the average ice mass loss over the entire ice sheet between 2002 and 2015 was estimated to be approximately 238 gigatons per year (Gt/yr) [5,6,7]. Meehl et al. indicated that global sea level would rise by approximately 6 m if Greenland’s ice sheet were to melt completely [8], and projected a sea level rise of 0.5 to 1.5 m by 2100 [9,10]. Monitoring these rapid changes in the polar regions is therefore essential for assessing cryospheric vulnerability and constraining regional and global climate models.

Due to the vast spatial extent and remoteness of polar regions, assessing rapid surface changes, such as glacier motion, relies heavily on remote sensing observations. One of the key technologies for cryospheric monitoring is synthetic aperture radar (SAR), which provides weather-independent observations with high spatial resolution (typically 1–100 m, depending on the acquisition mode). Interferometric SAR (InSAR) has proven to be a powerful tool for monitoring glacier flow [11,12,13,14,15] and for detecting crustal uplift adjacent to melting glaciers and ice sheets, which can be used to estimate ice mass loss [16,17,18]. However, the quality of InSAR measurements is often limited by temporal and volumetric decorrelation arising from surface changes between SAR acquisitions.

The quality of InSAR measurements is commonly evaluated using interferometric coherence, which quantifies the degree of correlation between two SAR observations. Coherence depends on several factors, including surface type, perpendicular baseline, temporal baseline, polarization, acquisition geometry, and so on. To date, coherence analyses have been conducted across various environments, including arid regions (e.g., volcanoes), vegetated areas, urban environments, agricultural regions, and wetlands [19,20,21,22,23,24]. However, despite recent progress, the coherence characteristics over snow- and ice-covered surfaces remain poorly understood, particularly across different radar frequencies, polarization modes, and cryospheric surface conditions. Recent studies have begun to clarify coherence behavior over snow- and ice-covered surfaces, showing that it is strongly modulated by radar wavelength, snow/firm stratigraphy, melt–refreeze processes, and glacier motion. In addition, interferometric coherence has emerged as a useful signal for a wide range of cryospheric applications, including glacier delineation, surge detection, meltwater–lake monitoring, snow/firm penetration analysis, and snow mass retrieval [25,26,27,28,29,30,31,32].

In this study, we evaluate the interferometric coherence of fully polarimetric (quad-pol) X-band TerraSAR-X (TSX) and L-band ALOS PALSAR data acquired over snow- and ice-covered regions. We primarily focus on the TSX satellite, which is currently the only spaceborne X-band SAR system capable of acquiring quad-pol data [33,34]. Because the short wavelength of X-band SAR (~3 cm) is highly sensitive to decorrelation effects, low coherence would generally be expected over rapidly changing snow- and ice-covered surfaces. Therefore, evaluating coherence levels using quad-pol TSX observations is particularly valuable for identifying the most suitable polarization for X-band InSAR applications in cryospheric regions.

## 2. Interferometric Coherence

Interferometric coherence represents the magnitude of the correlation between two SAR observations, $s_{1}$ and $s_{2}$. Coherence is calculated using the following equation:

(1) $$\gamma =\frac{\left|\langle s_{1}s_{2}^{*}\rangle \right|}{\sqrt{\langle s_{1}s_{1}^{*}\rangle \langle s_{2}s_{2}^{*}\rangle }},$$

where $s_{1}$ and $s_{2}$ denote the complex pixel values of two single-look complex (SLC) SAR images, s* represents the complex conjugate of s, and $\langle \cdot \rangle$ denotes the ensemble average over adjacent pixels. In theory, perfectly correlated SAR images yield a coherence value of 1, whereas a coherence value of 0 indicates no correlation between the two SAR images [20,35,36,37]. Coherence is therefore widely used as an indicator of the quality of an InSAR interferogram pair.

Coherence reflects contributions primarily from spatial, temporal, and thermal decorrelation processes [19,35,36], and is commonly expressed as

(2) $$\gamma =\gamma_{spatial}\cdot \gamma_{temporal}\cdot \gamma_{thermal},$$

where γ is the total coherence, and $\gamma_{spatial}$, $\gamma_{temporal}$, and $\gamma_{thermal}$ represent spatial, temporal, and thermal decorrelation components, respectively. Thermal coherence ($\gamma_{thermal}$) is related to the signal-to-noise ratio (SNR) of the radar signal [36]. In this study, $\gamma_{thermal}$ is assumed to be unity, as the SNR of most spaceborne SAR systems is sufficiently high [20,36,38].

Temporal decorrelation ($\gamma_{temporal}$) reflects coherence loss caused by surface changes occurring between two SAR acquisitions. Areas dominated by volume scattering, such as forests, wetlands, and snow- or ice-covered regions, typically exhibit stronger temporal decorrelation than areas dominated by surface or double-bounce scattering, such as deserts and urban environments [20,24,38,39]. Spatial decorrelation, also known as baseline decorrelation, degrades spatial coherence ($\gamma_{spatial}$) in proportion to the perpendicular baseline separating the satellite acquisition positions [36,40]. This occurs because different viewing geometries yield distinct spatial-frequency spectra reflected from the target [41]. Spatial decorrelation effects are more pronounced in regions characterized by strong volume scattering, such as forests and glaciers, where SAR signal penetration depth is relatively large [20,38].

## 3. Study Area

**Figure 1:**
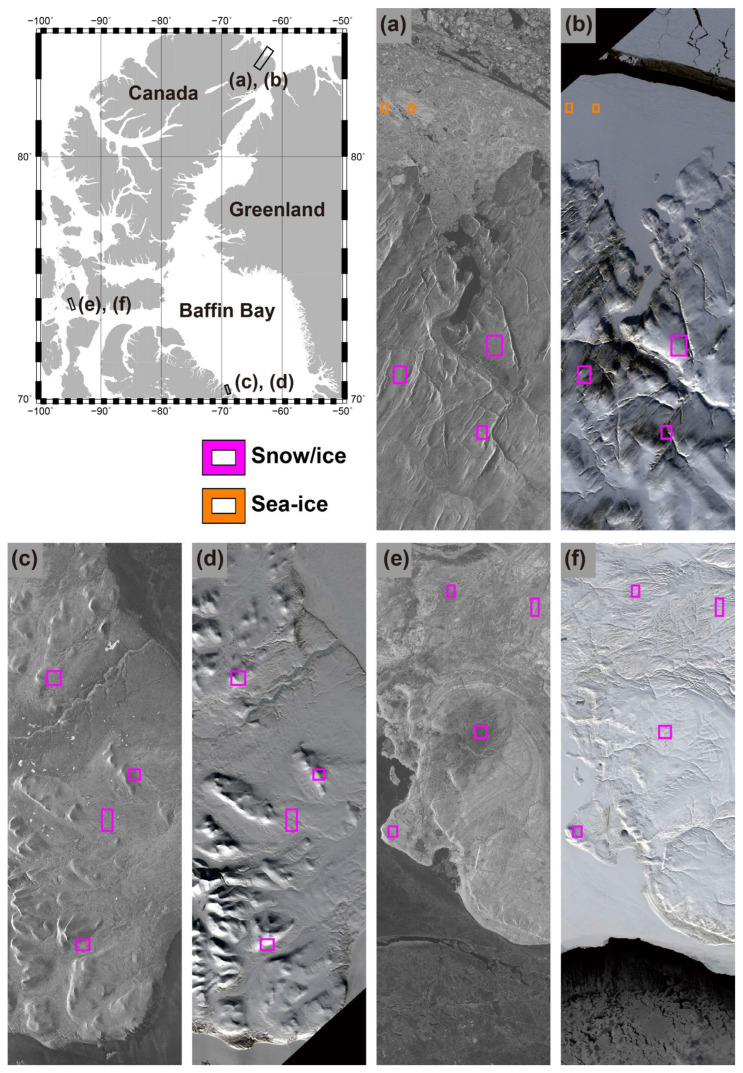
Location map showing the three TSX quad-pol swaths used in this study, marked by black frames. The remaining panels present SAR amplitude images (HH-pol) (**a**,**c**,**e**) and corresponding Landsat-8 Operational Land Imager (OLI) optical images (**b**,**d**,**f**) of the study areas in Canada, including Ellesmere Island (**a**,**b**), Baffin Island (**c**,**d**), and Cornwallis Island (**e**,**f**). Areas selected for quantitative coherence analysis over snow/ice-covered surfaces are indicated by magenta frames, while sea ice regions are highlighted by orange frames.

To evaluate the coherence characteristics of quad-polarimetric X-band TSX data over polar regions, three snow- and ice-covered study areas were selected, as shown in Figure 1. The selected areas are located in northeastern Canada, where large portions of the terrain are covered by glaciers and permanent ice. Both SAR amplitude images and optical images acquired by the Landsat-8 Operational Land Imager (OLI) are presented to illustrate surface conditions (Figure 1a–f). Most of the study sites are dominated by snow- or ice-covered surfaces, and the surrounding seas are covered by sea ice for much of the year. For coherence analysis, regions of interest (ROIs) representing snow- and ice-covered surfaces (magenta polygons) and sea ice (orange polygons) were selected based on visual interpretation in Figure 1b,d,f based on Landsat-8 OLI optical imagery and coherence maps. These ROIs were used to evaluate coherence characteristics as a function of surface type and polarization. In addition, a comparative coherence analysis was conducted using quad-pol X-band TSX (~3.1 cm) and L-band ALOS PALSAR (~23.0 cm) data over glacier- and snow-covered areas to examine whether quad-pol observations at different wavelengths exhibit similar coherence behavior. The Ellesmere Island region was selected for this comparison because both X- and L-band SAR observations were available over a similar geographic area, although the datasets were acquired at different times.

## 4. SAR Data and Processing

### 4.1. SAR Data

In this study, quad-polarimetric TSX data acquired in Dual Receive Antenna (DRA) mode were used. In DRA mode, TSX transmits the radar signal using the full antenna aperture and receives backscattered signals through two independent receiving channels. By electrically dividing the antenna into two sections, quad-pol SAR observations can be obtained. The DRA StripMap Campaign mode was conducted over a limited period between April and May 2010. Quad-pol SAR observations acquired in DRA mode have a swath width of approximately 15 km, which is half that of the standard StripMap swath (~30 km). The technical characteristics of the quad-pol TSX datasets used in this study are summarized in Table 1.

Two datasets were used to evaluate the coherence characteristics of quad-pol SAR images. The first dataset comprises 57 quad-pol TSX SAR images acquired along 20 swaths over northern polar regions covered by snow and/or ice during the DRA campaign conducted between April and May 2010. The temporal baselines of the interferometric pairs range from 11 to 22 days, which is sufficient to maintain relatively high coherence in short-wavelength X-band SAR observations. The absolute perpendicular baselines range from 12 to 226 m. For L-band comparison, three interferometric ALOS PALSAR pairs were available over the Ellesmere Island site, acquired on 29 March, 14 May 2007, and 19 November 2009. This limitation is due to the ALOS observation strategy, which did not prioritize polarimetric SAR acquisitions over polar regions. The interferometric pair has a temporal baseline ranging from 46 to 654 days, corresponding to the ALOS satellite revisit cycle, and a perpendicular baseline ranging from 30 to 260 m, which is sufficiently small to minimize spatial decorrelation effects.

### 4.2. Coherence Calculation

For each interferometric pair, four polarimetric interferograms were generated using the GAMMA software package [42]. An interferogram is a complex-valued image calculated by multiplying one SAR image by the complex conjugate of the other SAR image. Interferometric coherence was estimated from the interferogram and the intensities of both SAR images. All interferograms were generated using a multilooking process with 16 independent pixels (4 looks in azimuth and 4 in range, corresponding to an approximate ground resolution of 4 m × 10 m for the TSX dataset), and the data were weighted for coherence estimation. Coherence analyses based on Equation (1) were performed using a 5 × 5 pixel estimation window, a commonly adopted approach for coherence calculation. Range spectral filtering was applied prior to coherence estimation to reduce spatial decorrelation effects [42]. The averaged coherence in each selected region was calculated to identify polarimetric differences. Since this study focuses on the overall coherence response of snow- and ice-covered surfaces rather than site-specific characteristics, averaged coherence values were adopted to provide representative estimates for each surface type.

**Figure 2:**
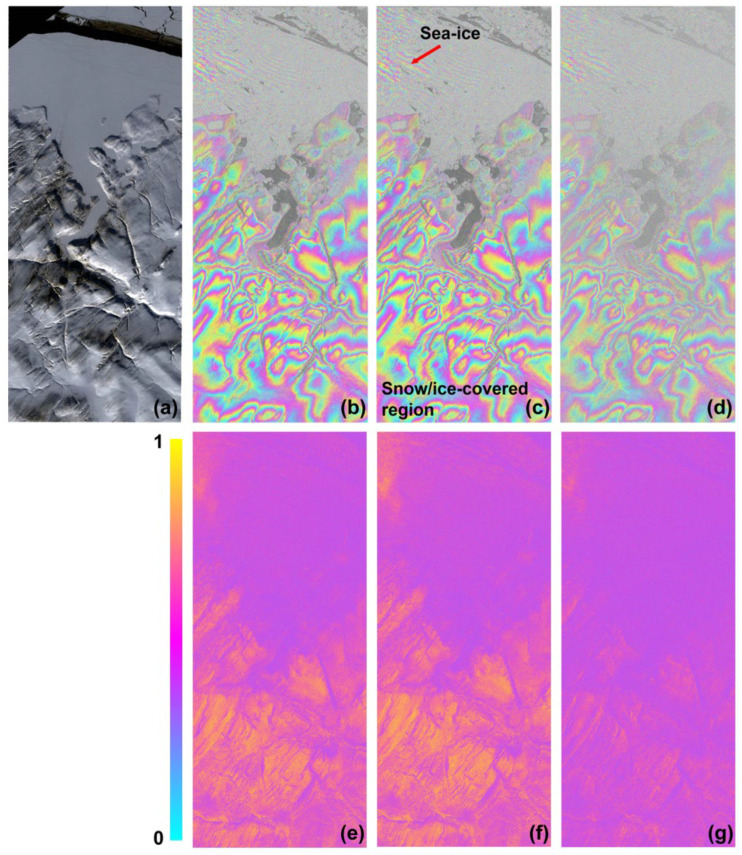
Landsat-8 OLI image (**a**) and TerraSAR-X (TSX) quad-pol interferograms (**b**–**d**) showing phase variations over snow/ice-covered terrain on Ellesmere Island, Canada, and offshore sea ice. The interferometric pair was acquired on 11 April 2010 and 22 April 2010 (11-day temporal baseline) with a perpendicular baseline of −45 m. The study area is located in Figure 1b. The three interferograms exhibit very similar fringe patterns. The co-polarized HH and VV interferograms (**b**,**c**) maintain higher coherence (**e**,**f**) and show clearer fringes than the cross-polarized HV interferogram (**d**,**g**).

For each interferometric pair, three interferograms were generated, corresponding to the HH, VV, and HV polarimetric components. For the first dataset, a total of 162 interferograms were produced, including 54 interferograms for each polarization. Across all interferometric pairs, the three polarimetric interferograms exhibit very similar fringe patterns, largely independent of polarization. Figure 2 presents an example of quad-pol, unfiltered, flat-earth-corrected interferograms acquired over Ellesmere Island, Canada, showing highly coherent interferometric phase over snow- and ice-covered surfaces and over sea ice. Only the HV-polarization interferogram is shown, as the HV and VH scattering matrix elements are theoretically identical under the assumption of reflection symmetry in polarimetric scattering theory [43]. Notably, coherence is well preserved over sea ice, indicating minimal surface motion during the 11-day temporal baseline between the two SAR acquisitions. Although quad-pol interferograms display similar fringe patterns, the coherence magnitudes vary across polarization channels. In particular, the co-polarized interferograms (Figure 2b,c,e,f) demonstrate that X-band SAR observations with a short temporal baseline (e.g., 11 days) can provide high-quality InSAR measurements. Moreover, the cross-polarized HV interferogram (Figure 2d,g) also maintains relatively high coherence values (>0.35). The noisier appearance of Figure 2d,g can be explained by the lower coherence and weaker backscattered power generally associated with the cross-polarized HV channel. As a result, the image exhibits a less distinct interferometric pattern and a more speckled texture compared with co-polarized interferograms.

## 5. Results

**Figure 3:**
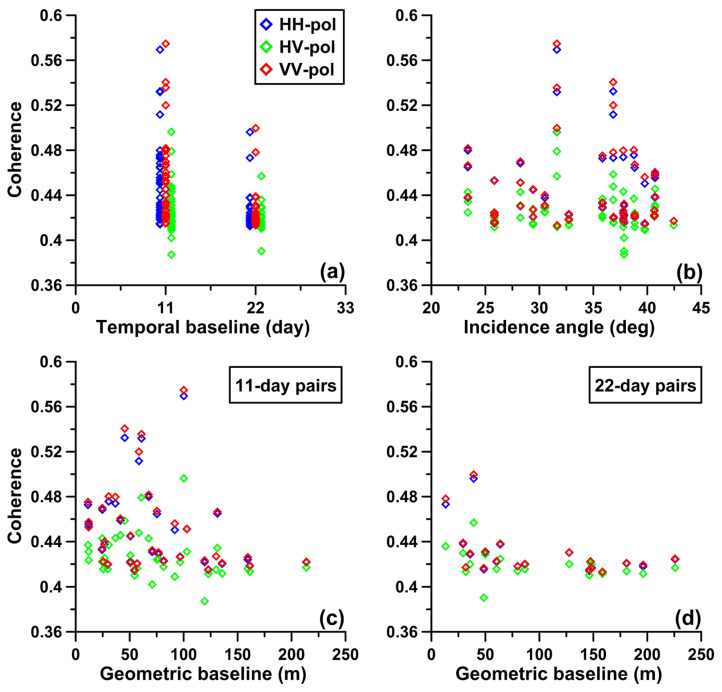
(**a**) Coherence as a function of temporal baseline. Temporal decorrelation is pronounced, although the available dataset includes only 11- and 22-day temporal baselines. (**b**) Coherence as a function of incidence angle. (**c**,**d**) Coherence as a function of perpendicular (geometric) baseline for interferometric pairs with (**c**) 11-day and (**d**) 22-day temporal baselines. Short perpendicular baselines (<100 m) generally yield relatively high coherence. Overall, no strong correlation is observed between coherence and these parameters. The co-polarized interferograms (HH and VV) exhibit substantially higher coherence than the cross-polarized interferograms (HV). Among the co-pol channels, VV shows the highest coherence in the polar region.

We first investigated the overall coherence characteristics of the TSX datasets over snow- and ice-covered areas by examining potential relationships between coherence and the temporal, perpendicular, and incidence angles (Figure 3). To isolate the contribution of the perpendicular baseline to coherence, coherence was analyzed separately for the 11- and 22-day temporal baselines. The results indicate relatively high coherence levels across all polarization channels, ranging from 0.38 to 0.57, with the highest values in VV polarization, followed by slightly lower values in HH polarization, and the lowest in HV polarization. These findings are consistent with previous studies reporting an inverse relationship between interferometric coherence and temporal baseline in natural environments [24,39,44]. However, the observation that VV polarization exhibits slightly higher coherence than HH polarization is noteworthy, as HH polarization typically yields the highest coherence in many other environments [44]. Snow- and ice-covered surfaces are generally associated with rapid temporal decorrelation over short time intervals of several days [45,46]. Therefore, it is notable that short-wavelength X-band SAR observations maintain relatively high coherence over snow- and ice-covered regions, particularly for short temporal baselines (Figure 3a). The analysis does not reveal a strong correlation between coherence and incidence angle (Figure 3b) or between coherence and perpendicular baseline (Figure 3c,d). Nevertheless, interferometric pairs with shorter perpendicular baselines (<100 m) tend to exhibit higher coherence than those with larger perpendicular baselines. To further investigate polarization-dependent coherence behavior, coherence was analyzed across three selected regions (small inset boxes in Figure 1) using six interferometric pairs with an 11-day temporal baseline to minimize temporal decorrelation. The resulting coherence values confirm that VV polarization generally exhibits slightly higher coherence (0.16–0.62) than HH polarization (0.16–0.60), while HV polarization shows lower coherence values ranging from 0.13 to 0.36.

**Figure 4:**
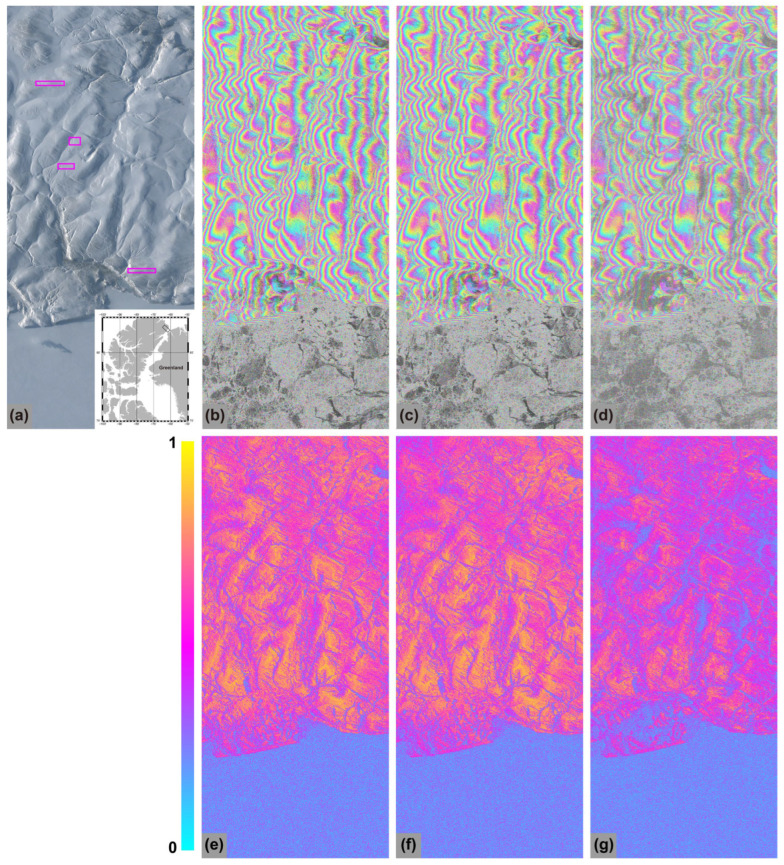
Landsat-8 OLI image (**a**) and ALOS quad-pol interferograms (**b**–**d**) over snow/ice-covered terrain on Ellesmere Island, Canada (see Figure 1a,b). The interferometric pair was acquired on 29 March 2007 and 14 May 2007 (temporal baseline: 46 days; perpendicular baseline: −236 m). The snow/ice-covered regions selected for coherence analysis are outlined by magenta boxes (**a**). The HH (**b**,**e**), VV (**c**,**f**), and HV (**d**,**g**) interferograms and coherences exhibit very similar fringe patterns with comparable coherence levels over the snow/ice-covered areas. Notably, the HV interferogram (**d**,**g**) retains low coherence relative to the co-polarized interferograms (**b**,**c**,**e**,**f**). Owing to temporal decorrelation over the 46-day interval, coherence is not preserved over the sea ice regions.

**Figure 5:**
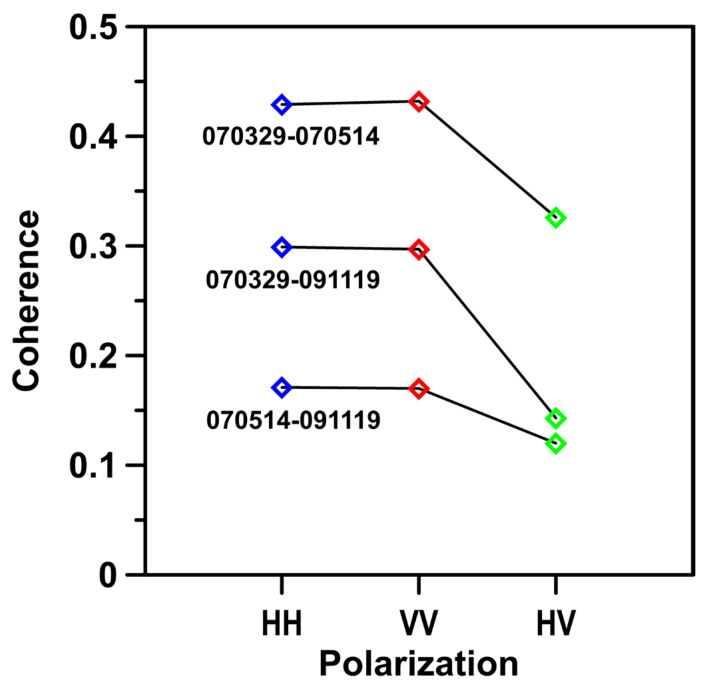
Coherence analysis over snow/ice-covered regions using ALOS quad-pol L-band SAR observations. The HH (blue) and VV (red) channels exhibit very similar coherence levels, whereas the HV (green) channel shows the lowest coherence.

We additionally analyzed coherence over snow- and ice-covered areas using L-band ALOS PALSAR observations (Figure 4a). Four snow- and ice-covered regions were selected for coherence estimation. The three polarization channels exhibit similar fringe patterns, with high coherence over snow- and ice-covered land surfaces and negligible coherence over sea ice areas. These results suggest that longer-wavelength L-band SAR observations maintain comparable coherence levels over snow- and ice-covered terrain regardless of polarization, whereas coherence over sea ice is largely lost due to surface motion during the 46-day temporal baseline (Figure 4b–g). Coherence analysis over smaller selected regions further shows similar coherence levels for HH and VV polarizations, both of which are slightly higher than those observed for HV polarization (Figure 5). Although the HV interferogram still shows recognizable interferometric structure in some areas, the quantitative coherence analysis indicates that the HH and VV channels maintain higher coherence than HV over the selected snow/ice-covered regions.

## 6. Discussion

The coherence analysis over snow- and ice-covered areas revealed that VV polarization exhibits higher coherence values than HH polarization. Insight into this observed behavior can be obtained by examining and comparing the polarimetric SAR amplitude characteristics over the same surface types. In general, VV-polarized backscattering is stronger than that of other polarizations over surfaces with high dielectric constants, such as water bodies [47]. Consistently, the polarization ratio between HH and VV backscattering is typically lower than unity over ocean surfaces and wet sea ice [48]. Because snow- and ice-covered surfaces, as well as sea ice, generally exhibit higher dielectric constants than dry urban or vegetated surfaces, stronger VV-polarized backscattering can be expected in these environments. Although snowfall, melt–refreeze processes, precipitation, and wind-driven surface changes may contribute to temporal decorrelation, we focused on direct comparisons between different polarimetric phases under the same meteorological conditions. Our study is based solely on TerraSAR-X observations; therefore, the generality of X-band coherence behavior remains to be further validated. Additional comparison with other X-band SAR systems, such as COSMO-SkyMed, will not be available because COSMO-SkyMed does not provide full polarimetric observations.

The relatively higher coherence in VV polarization over snow/ice-covered surfaces may suggest that VV is more closely associated with a stable surface or near-surface scattering component, whereas HH is more sensitive to variations in surface roughness, snow/ice structure, and volume scattering within the snowpack [27,49]. Differences in scattering characteristics may lead to greater fluctuations in the HH scattering phase center between repeat radar acquisitions, thereby reducing coherence [27,49]. In addition, polarization-dependent propagation through dry snow can be affected by dielectric anisotropy, which may further shift the effective phase center and contribute to the observed coherence differences between HH and VV polarizations [50].

**Figure 6:**
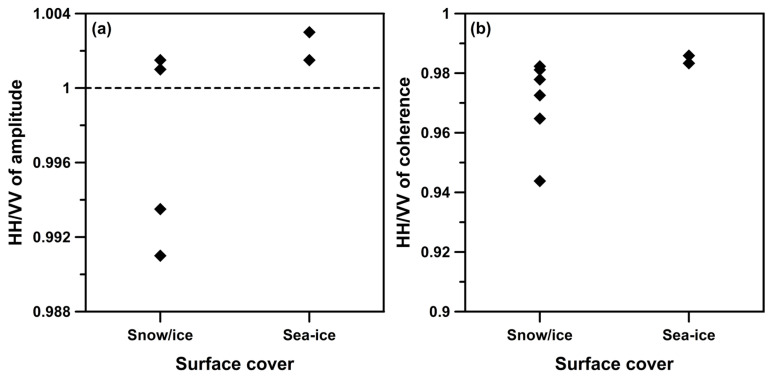
Co-polarization ratio (HH/VV) of amplitude (**a**) and coherence (**b**) over selected snow/ice-covered and sea ice regions. The amplitude ratios (**a**) indicate similar values for HH and VV, with VV being slightly higher in both snow/ice-covered and sea ice areas. The coherence ratios (**b**) show higher VV coherence than HH over both snow/ice-covered and sea ice regions.

To investigate the relationship between interferometric coherence and backscattering strength, polarimetric SAR amplitude values were calculated for selected snow- and ice-covered regions and compared with the corresponding polarimetric coherence values (Figure 6). Prior to averaging, a 5 × 5 pixel moving window, consistent with the coherence estimation window, was applied to reduce speckle noise. In addition, a Gaussian weighting function was applied to further suppress speckle. Mean VV- and HH-polarized amplitude values were then computed, and the HH/VV amplitude ratio was derived (Figure 6a). The HH/VV amplitude ratio provides a normalized measure of the relative backscattering strength between HH and VV polarizations, independent of the absolute amplitude values. Similarly, the HH/VV coherence ratio was calculated to enable direct comparison with the amplitude ratio (Figure 6b).

The HH/VV amplitude and coherence ratios allow a quantitative assessment of the relationship between polarimetric backscattering and coherence characteristics (Figure 6). The HH/VV amplitude ratios indicate that snow- and ice-covered surfaces and sea ice regions exhibit nearly equal HH- and VV-polarized backscattering amplitudes (HH/VV ≈ 1). In contrast, the HH/VV coherence ratios are consistently lower than unity (0.94–0.98), indicating higher coherence in VV polarization than in HH polarization over these surfaces (Figure 6b). A positive correlation between the HH/VV amplitude ratio and the HH/VV coherence ratio is observed for snow- and ice-covered regions.

Coherence analyses of quad-polarimetric X-band (3.1 cm) and L-band (23.0 cm) SAR data reveal wavelength-dependent coherence behavior over snow- and ice-covered areas. While VV polarization exhibits higher coherence than HH polarization in X-band observations, HH and VV coherence values are comparable in L-band data. This difference is likely related to wavelength-dependent penetration depth and scattering mechanisms in snow and ice. The short-wavelength X-band SAR signal predominantly interacts with the upper layers of snow- and ice-covered surfaces and sea ice, which are relatively heterogeneous and sensitive to temperature variations and wind-driven surface changes. In contrast, the longer-wavelength L-band SAR signal can penetrate deeper into snow and ice and may interact with more stable subsurface layers or, in some cases, with the underlying bedrock. The longer wavelength in the L-band enables deeper penetration into snow and firn, increasing the contribution of subsurface and volume scattering compared to shorter wavelengths [51]. Therefore, L-band coherence is likely to be more strongly controlled by penetration depth and internal snowpack heterogeneity, so its polarization dependence may differ from that observed at X-band [27,49,51].

According to scattering theory, short-wavelength SAR signals are generally more susceptible to temporal and volume decorrelation [43,52], and low coherence is therefore often expected over snow- and ice-covered regions. Indeed, X-band SAR observations have often been reported to suffer from strong temporal and surface decorrelation in interferometric applications over snow- and ice-covered areas [45,53,54]. However, one key finding of this study is that X-band TSX observations can yield highly coherent interferograms over snow- and ice-covered regions when short temporal baselines and well-controlled orbital geometries are used. Given that high coherence can be achieved at X-band with short temporal baselines, longer-wavelength SAR systems such as Sentinel-1 C-band (12-day revisit cycle) and ALOS-2 L-band (14-day repeat pass) are expected to provide even higher interferometric coherence over polar regions. Furthermore, SAR constellations capable of acquiring data with very short temporal separations, such as TanDEM-X (second-scale baseline) and COSMO-SkyMed (daily revisit), are particularly well suited for monitoring sea ice dynamics and rapid surface changes in polar environments.

A limitation of this study is that the observed coherence difference between HH and VV is generally modest and, therefore, should not be interpreted as evidence of a strong superiority of VV polarization. The results indicate only a slight, recurrent tendency for VV to exhibit higher coherence than HH in the analyzed TerraSAR-X dataset over snow- and ice-covered surfaces. Nevertheless, this tendency may still provide useful practical guidance for ground-based radar systems that can select a polarization channel, since even a small coherence advantage can be beneficial for interferometric observation.

Another limitation of this study is the absence of independent cross-validation data. A full cross-validation using independent multi-source datasets was not possible because comparable quad-pol X-band observations acquired over the same snow- and ice-covered regions, during the same period, and under similar acquisition geometries were not available. Therefore, the observed polarization-dependent coherence characteristics should be interpreted within the constraints of the available TerraSAR-X dataset, rather than as a universally applicable conclusion. Nevertheless, the interpretation was supported by consistency checks across different acquisition pairs, comparisons of coherence patterns across polarization channels, and a physical interpretation based on known scattering mechanisms on snow- and ice-covered surfaces. The main contribution of this study lies in the systematic observational assessment of polarization-dependent X-band coherence and backscattering characteristics over snow- and ice-covered regions, rather than in the development of a new interferometric processing algorithm. Although the causal mechanisms underlying the observed VV–HH coherence differences cannot be fully isolated from the available data alone, the results suggest that differences in surface-scattering stability, roughness sensitivity, volume-scattering contributions, and polarization-dependent phase-center variations may contribute to the observed behavior. Further studies using independent datasets and physically based scattering models are needed to validate these findings and clarify the underlying mechanisms.

## 7. Conclusions

We conducted a coherence analysis of quad-polarimetric X-band TSX observations acquired during the DRA campaign in 2010 and found that VV-polarized interferograms exhibit higher coherence than HH-polarized interferograms over snow- and ice-covered regions. In contrast, ALOS PALSAR L-band observations show very similar coherence levels for both co-polarized channels. Coherent interferometric phases were well preserved over most snow- and ice-covered regions when the temporal baseline was 11 days. Although some interferometric pairs with a 22-day temporal baseline also exhibited moderate coherence values (>0.4), the coherence levels were significantly lower than those observed for the 11-day interferograms. Our analysis further indicates that smaller perpendicular baselines (<100 m) generally result in higher coherence over snow- and ice-covered regions. These results suggest that optimal interferometric conditions for monitoring snow- and ice-covered regions using TSX data involve VV-polarized acquisitions with short temporal baselines and small perpendicular baselines.

## Figures and Tables

**Table 1:** Characteristics of TSX SAR Data.

Parameter	TerraSAR-X
Carrier frequency	X-band (9.6 GHz)
Wavelength	3.1 cm
Polarization	Quad-pol
Revisit cycle	11 days
Pulse repetition frequency	2915–3197 Hz
ADC sampling rate	164.8 MHz
Azimuth pixel spacing	2.35~2.43 m
Range pixel spacing	0.91 m
Pulse repetition frequency	2915–3197 Hz

## Data Availability

The original contributions presented in this study are included in the article. For further information, please contact the corresponding author.
